# The influence of male and female overweight/obesity on IVF outcomes: a cohort study based on registration in Western China

**DOI:** 10.1186/s12978-022-01558-9

**Published:** 2023-01-02

**Authors:** Xiang Liu, Shengjia Shi, Jianhua Sun, Yuan He, Zhou Zhang, Junping Xing, Tie Chong

**Affiliations:** 1 The Second Affiliated Hospital of Xi’an Jiao Tong University, Xi’an, 710004 Shaanxi China; 2 Reproductive Center of Northwest Women’s and Children’s Hospital, Xi’an, 710061 Shaanxi China; 3 The First Affiliated Hospital of Xi’an Jiao Tong University, Xi’an, 710061 Shaanxi China

**Keywords:** Overweight/obesity, BMI, In vitro fertilization-embryo transfer (IVF-ET), CPR, LBR

## Abstract

**Background:**

Overweight/obesity can affect fertility, increase the risk of pregnancy complications, and affect the outcome of assisted reproductive technology (ART). However, due to confounding factors, the accuracy and uniformity of published findings on IVF outcomes have been disputed. This study aimed to assess the effects of both male and female body mass index (BMI), individually and in combination, on IVF outcomes.

**Methods:**

This retrospective cohort study included 11,191 couples undergoing IVF. Per the Chinese BMI standard, the couples were divided into four groups: normal; female overweight/obesity; male overweight/obesity; and combined male and female overweight/obesity. The IVF outcomes of the four groups were compared and analysed.

**Results:**

Regarding the 6569 first fresh IVF-ET cycles, compared with the normal weight group, the female overweight/obesity and combined male/female overweight/obesity groups had much lower numbers of available embryos and high-quality embryos (p < 0.05); additionally, the fertilization (p < 0.001) and normal fertilization rates (p < 0.001) were significantly decreased in the female overweight/obesity group. The combined male/female overweight/obesity group had significant reductions in the available embryo (p = 0.002), high-quality embryo (p = 0.010), fertilization (p = 0.001) and normal fertilization rates (p < 0.001); however, neither male or female overweight/obesity nor their combination significantly affected the clinical pregnancy rate (CPR), live birth rate (LBR) or abortion rate (p > 0.05).

**Conclusion:**

Our findings support the notion that overweight/obesity does not influence pregnancy success; however, we found that overweight/obesity affects the fertilization rate and embryo number and that there are sex differences.

## Introduction

Overweight/obesity is an important global public health problem because it has not only a negative impact on quality of life but also a severe impact on health [1]. From 1979 to 2016, the number of adult obese women worldwide increased from 69 to 390 million, and the number of adult obese males increased from 31 to 281 million [2]. With its large population base and rapid growth rate, China has the world’s highest number of obese people [3]. According to the “Report on Nutrition and Chronic Disease Status of Chinese Residents (2020)” issued by the State Council of China on December 23, 2020, approximately 600 million people in China are overweight/obese, with an overweight rate of 34.3% and an obesity rate of 16.4% among adults. Body mass index (BMI) is the key indicator for measuring overweight/obesity, and based on data from European and American populations, the World Health Organization (WHO) established an international standard, wherein the normal BMI range is 18.5–24.9 kg/m^2^, a BMI of 25–29.9 kg/m^2^ indicates overweight, and a BMI ≥ 30 kg/m^2^ indicates obesity. As there are racial differences between Asian populations and European and American populations, the BMI standard of the WHO is not suitable for Chinese people [4]. For this reason, the BMI reference standard for Chinese adults is as follows: 18.5–23.9 kg/m^2^ is the normal range, 24–27.9 kg/m^2^ indicates overweight, and ≥ 28 kg/m^2^ indicates obesity [5].

Overweight/obesity can increase the incidences of many diseases, and their onset and progression are closely linked to those of endocrine diseases, which can affect the functions of multiple human systems, such as the reproductive system [6]. As a result, obesity is a risk factor for menstrual disorders and ovulation disorders in women, impairs sperm function and increases the risk of erectile dysfunction in men, thereby reducing fertility in couples [7–9].

Most infertile couples need to use ART to meet their fertility needs, and in vitro fertilization–embryo transfer (IVF–ET) is one of the most common forms of ART. It is estimated that by 2100, the percentage of people born by ART may reach 1.4–3.5% of the global population (approximately 157–394 million people); more than 2 million treatment cycles of IVF and intracytoplasmic sperm injection (ICSI) are completed every year [10]. Increasing evidence suggests that female overweight/obesity can negatively affect ART outcomes. The influence of obesity on the outcomes of ART exceeds that of being overweight and has a dose–response relationship [1, 11–14]. Similarly, several studies based on large populations have found that male overweight/obesity can negatively affect semen parameters and thereby reduce the potential for male fertility [15–19]. However, there is no consistent conclusion regarding the impact of overweight/obesity on embryonic parameters and the pregnancy outcomes of ART, especially IVF [18, 20–24]; therefore, more robust research is necessary. Given that the pregnancy success rate of IVF–ET depends on the health of both members of the couple, it is necessary to evaluate the combined influence of male and female BMI on embryo quality and the pregnancy outcome of IVF treatment. Of the last two retrospective studies evaluating the impact of male and female BMI on the clinical pregnancy rate (CPR) and the live birth rate (LBR), one found that individual or combined male and female overweight/obesity has a negative impact on the LBR after IVF treatment [25]. Another study concluded that there was no significant correlation between male or female overweight and the fertilization rate, embryo score, CPR or LBR [21]. The conclusions of these studies were limited by confounding factors that affected the results of the analysis, such as a small sample size and the inclusion of subjects who smoked; other associated confounding factors were not considered in these studies, and the study populations comprised mainly European or American participants. Therefore, in further research, in addition to considering the above influencing factors and adopting standardized methods that are suitable for measuring the BMIs of individuals of different races, combined male and female BMI and IVF cycle characteristics, including the number of oocytes harvested, endometrial thickness, IVF-ET cycle, number of transferred embryos and embryo quality, should be considered comprehensively.

Although China has the highest incidence of overweight/obese individuals, data on the IVF outcomes of Chinese overweight/obese populations are scarce. Therefore, we studied the effects of BMI on IVF outcomes among Chinese couples. Under the premise of excluding confounding factors that affect IVF outcomes, we systematically reviewed and summarized the data of patients registered as overweight/obesity to investigate the impacts of both male and female BMI, individually and in combination, on the fertilization rate, embryo score, and incidences of clinical pregnancies and live births among couples undergoing IVF treatment.

## Materials and methods

### Study period and participants

After obtaining approval from the Northwest Women and Children's Hospital, we conducted a prospective cohort study with all couples undergoing their first fresh IVF cycles from 01/2015–12/2020 at the Reproductive Center of Northwest Women and Children's Hospital, for whom male/female weight and height information were available. If the couple was interested in participating, a member of the study team discussed the study in detail and obtain written informed consent prior to the start of the IVF cycle or early in the cycle. Couples were selected according to the following criteria: (1) were undergoing their first fresh oocyte retrieval cycle; (2) had regular menstrual cycles; (3) had ≥ 4 oocytes harvested; (4) were aged between 22 and 36 years; (5) had normal chromosomes; and (6) had no reproductive tract infections, such as mycoplasma, chlamydia or gonorrhoea, in the past three months. The subjects also had none of the following conditions: (1) polycystic ovary syndrome (PCOS); (2) hyperprolactinemia; or (3) diabetes or other diseases that may affect IVF outcomes.

### Body mass index assessment

**Fig. 1:**
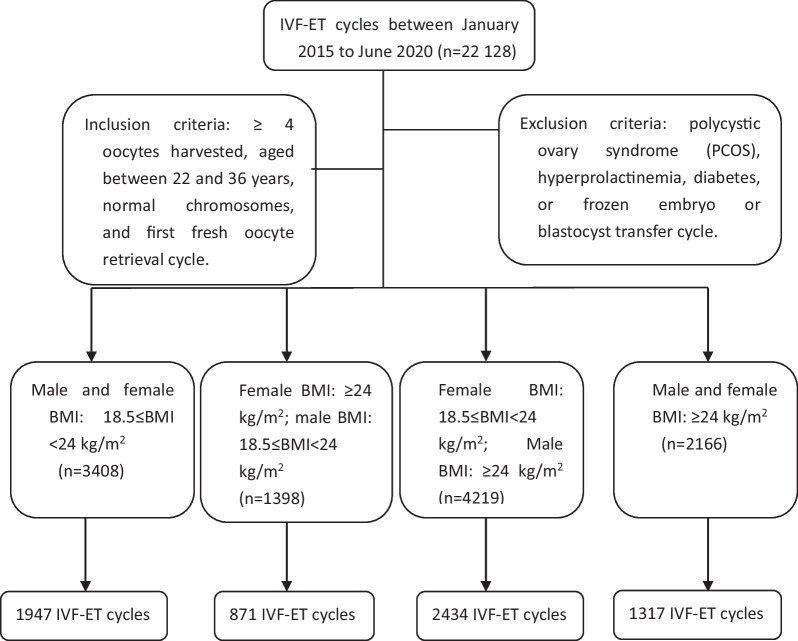
Flow chart of the study cohort characteristics

BMI assessment data were abstracted from electronic medical records and patient medical charts. Upon presenting at the Reproductive Center of Northwest Women and Children's Hospital for infertility evaluation, male/female height and weight were measured by medical assistants using standardized protocols per clinical practice. According to the obesity and overweight standards of the Chinese Obesity Working Group, couples were divided into 4 groups: Group 1 (n = 3408, male and female BMI: 18.5 ≤ BMI < 24 kg/m^2^); Group 2 (n = 1398, female BMI: ≥ 24 kg/m^2^; male BMI: 18.5 ≤ BMI < 24 kg/m^2^); Group 3 (n = 4219, female BMI: 18.5 ≤ BMI < 24 kg/m^2^; male BMI: ≥ 24 kg/m^2^); and Group 4 (n = 2166, female BMI: ≥ 24 kg/m^2^; male BMI ≥ 24 kg/m^2^) (Fig. 1).

### Research method

On the second day after oocytes were harvested, fertilization was judged by the presence of two pronuclei (2PN). If 2PN was displayed, fertilization was considered to have occurred. After fertilization, the cells were cultured in vitro for 72 h (cleavage stage) or 120 h (blastocyst stage) and transferred according to the number and quality of the embryos/blastocysts on that day and the woman’s progesterone value. This study included women who underwent embryo/blastocyst transfer. Beginning on the day of embryo/blastocyst transfer, 60 mg of progesterone (Zhejiang Xianju, China) was injected intramuscularly daily until day 70 or after the achievement of a clinical pregnancy. A biochemical pregnancy was defined as detectable human chorionic gonadotropin (hCG) in blood samples at 12 to 14 days after ET, and a clinical pregnancy was defined as one or more gestational sacs by B ultrasound at 4 to 6 weeks after ET.

### Statistical analysis

Continuous variables are presented as the mean and standard deviation (SD), and categorical variables were described with the chi-square test. Analysis of variance was used for comparisons between groups, and for p ≤ 0.05, the least significant difference (LSD) t test was used to compare groups, with Group 1 as the reference. A linear regression model was employed to assess the associations of BMI status with the individual available embryo rate, high-quality embryo rate, fertilization rate and cleavage rate by calculating the β-coefficient and 95% confidence interval (CI). The odds ratio (OR) and 95% CI were calculated using a multivariate logistic regression model. Model 1 was adjusted for male and female age. Model 2 was adjusted for female age, male age, infertility duration, the number of available embryos, the left testicular volume, the right testicular volume, and the oestradiol (E2), follicle-stimulating hormone (FSH), progesterone (P), and luteinizing hormone (LH) levels on the day that the hCG level was measured. Two-sided p values less than 0.05 were considered significant. All analyses were conducted using SAS 9.4.

## Results

A total of 11,191 couples with 6569 first fresh embryo transfer cycles were analysed; we included only the first IVF treatment cycle. The numbers of first fresh IVF–ET cycles in Groups 1, 2, 3 and 4 were 1947, 871, 2434, and 1317, respectively.

Table 1 shows the data on the basic characteristics of the subjects.

**Table 1:** Demographics of study samples by male and female BMI status

Variables	Group 1 (N = 3408)	Group 2 (N = 1398)	Group 3 (N = 4219)	Group 4 (N = 2166)
Male age (years)	30.21 ± 3.06	30.29 ± 2.98	30.56 ± 2.99*	30.58 ± 2.99*
Female age (years)	29.07 ± 2.93	28.96 ± 3.07	29.47 ± 2.95*	29.36 ± 2.99*
Endometrial thickness (mm)	11.67 ± 2.31	11.85 ± 4.05	11.68 ± 2.74	11.76 ± 3.04
Infertility duration (years)	3.17 ± 1.99	3.48 ± 2.05*	3.19 ± 1.99	3.59 ± 2.12*
E2 on HCG day (mIU/mL)	4814.92 ± 2761.74	3743.38 ± 2391.52*	4706.63 ± 2703.82	3817.43 ± 2450.54*
FSH on HCG day (mIU/mL)	18.13 ± 8.82	14.79 ± 7.29*	17.68 ± 7.87*	14.82 ± 6.18*
P on HCG day (mIU/mL)	1.40 ± 0.78	1.21 ± 0.74	1.69 ± 20.77	1.19 ± 0.68
LH on HCG day (ng/mL)	2.57 ± 44.44	2.76 ± 43.10	6.68 ± 187.17	1.62 ± 1.31
Number of available embryos	6.41 ± 3.88	6.00 ± 3.71*	6.35 ± 3.76	6.03 ± 3.87*
Number of high-quality embryos	3.98 ± 3.13	3.71 ± 3.02*	3.99 ± 3.11	3.77 ± 3.21*
Number of oocytes	12.07 ± 5.59	11.75 ± 5.53	11.94 ± 5.62	11.84 ± 5.79
Number of 2pn	7.58 ± 4.16	7.03 ± 4.00*	7.47 ± 4.01	7.17 ± 4.16*
Number of blastocysts	1.68 ± 2.83	1.75 ± 2.80	1.79 ± 2.81	1.64 ± 2.77
Number of transferred embryos (n, %)				
1	164 (17.58)	81 (18.93)	192 (16.90)	138 (20.78)
2	769 (82.42)	347 (81.07)	944 (83.10)	526 (79.22)
Number of transferred blastocysts (n, %)				
1	771 (76.04)	341 (76.98)	1005 (77.43)	509 (77.95)
2	243 (23.96)	102 (23.02)	293 (22.57)	144 (22.05)
Type of embryo transferred (n, %)				
Cleavage stage embryo	933 (47.92)	428 (49.14)	1136 (46.67)	664 (50.42)
Blastocyst	1014 (52.08)	443 (50.86)	1298 (53.33)	653 (49.58)
Quality o transferred cleavage embryos				
Number of high-quality embryos	1.47 ± 0.50	1.41 ± 0.49	1.42 ± 0.49	1.43 ± 0.50
Number of available embryos	1.83 ± 0.38	1.81 ± 0.39	1.83 ± 0.37	1.80 ± 0.40
Quality of transferred blastocysts				
Number of high-quality blastocysts	1.03 ± 0.16	1.03 ± 0.17	1.03 ± 0.17	1.04 ± 0.19
Number of available blastocysts	1.24 ± 0.43	1.23 ± 0.42	1.22 ± 0.42	1.22 ± 0.41
Left testicular volume (mL)	14.30 ± 1.96	14.27 ± 1.86	14.70 ± 2.23*	14.77 ± 2.40*
Right testicular volume (mL)	14.67 ± 20.67	14.29 ± 1.82	14.74 ± 2.20	14.76 ± 2.40
Number of oocytes harvested	12.07 ± 5.59	11.75 ± 5.53	11.94 ± 5.62	11.84 ± 5.79
Technicians title (Junior/intermediate/senior) (n)	2891/446/71	1204/166/28	3563/564/92	1820/295/51
Smoking (n, %)	585 (17.17)	258 (18.45)	792 (18.77)	411 (18.98)
2015–2019 IVF cycles (n)	604/707/788/921/388	221/268/330/381/198	708/851/930/1187/543	348/424/504/616/274

Group 1: male and female BMI, 18.5 ≤ BMI < 24 kg/m^2^. Group 2: female BMI, ≥ 24 kg/m^2^; male BMI, 18.5 ≤ BMI < 24 kg/m^2^. Group 3: female BMI, 18.5 ≤ BMI < 24 kg/m^2^; male BMI: ≥ 24 kg/m^2^. Group 4: male and female BMI, ≥ 24 kg/m^2^

The values are presented as the mean (± SD), and the least significant difference (LSD) *t*-test was used to compare groups 2–3 with group 1, respectively

*FSH* follicle-stimulating hormone; *HCG* human chorionic gonadotropin

^*^P < 0.05 was considered statistically significant

After the exclusion of patients with fewer than 4 harvested oocytes, there was no significant difference in the number of oocytes harvested among the four groups (p > 0.05), suggesting that when the number of oocytes harvested was ≥ 4, overweight/obesity did not affect the number of oocytes harvested during IVF. Although the left testicular volumes of the men in Group 3 and Group 4 were significantly different from those of the men in Group 1 (p < 0.05), their left testicular volumes were within the normal range (testicular volume ≥ 12 ml). Even when we restricted the ages of both spouses to between 22 and 36 years, the baseline distribution of the variables, including age, among the four groups was still unbalanced. For this reason, we established Model 2 to adjust for the variables contributing to the baseline imbalance.

Table 2 shows the IVF outcomes of the four groups with no adjustments for the baseline imbalances of male and female age or other variables. There were no significant differences in the available embryo rate, high-quality embryo rate, cleavage rate, implantation rate, CPR, abortion rate, or LBR (p > 0.05) among the four groups. Compared with those of Group 1, the fertilization rate and the normal fertilization rate of Groups 2 and 4 and the blastocyst rate of Group 3 were significantly different (p < 0.05).

**Table 2:** Comparison of clinical outcomes from IVF treatment between male and female BMI status

Variables	Group 1 (N = 3408)	Group 2 (N = 1398)	Group 3 (N = 4219)	Group 4 (N = 2166)	P-trend
Available embryo rate (%)	64.61 ± 22.83	63.68 ± 23.78	64.65 ± 22.95	63.41 ± 23.86	0.129
High-quality embryo rate (%)	39.80 ± 25.17	38.98 ± 26.10	40.34 ± 25.57	39.29 ± 26.32	0.279
Fertilization rate (%)	84.01 ± 17.42	81.46 ± 18.66*	84.31 ± 17.13	82.51 ± 18.17*	** < 0.0001**
Normal fertilization rate (%)	63.15 ± 20.30	60.36 ± 20.68*	63.33 ± 19.94	61.14 ± 20.83*	** < 0.0001**
Cleavage rate (%)	98.51 ± 5.12	98.51 ± 5.23	98.72 ± 4.26	98.44 ± 5.13	0.096
Blastocyst rate (%)	31.55 ± 32.69	33.14 ± 32.26	33.59 ± 32.94*	32.63 ± 32.44	0.085
Implantation rate (%)	86.73 ± 16.38	86.83 ± 17.27	87.33 ± 18.40	87.08 ± 17.59	0.482
Clinical pregnancy (n, %)	1274/1947 (65.43)	588/871 (67.51)	1619/2434 (66.52)	895/1317 (67.96)	0.349
Abortion (n, %)	154/1274 (12.09)	82/588 (13.95)	171/1619 (10.56)	124/895 (13.85)	0.593
Live birth (n, %)	1096/1947 (56.29)	501/871 (57.52)	1421/2434 (58.38)	757/1317 (57.48)	0.838

The values are presented as the mean (± SD) and proportion and were compared using linear regression and logistic regression, respectively (Group 1 as the reference)

^*^P < 0.05 was considered statistically significant, or marked in bold

Table 3 shows the IVF outcomes of the four groups after adjustments were made for the baseline imbalances in male and female age and other variables. Compared with those of Group 1, the available embryo rate, fertilization rate, and normal fertilization rate of Groups 2 and 4 were all significantly reduced (p < 0.05), and the high-quality embryo rate [adjusted β-coefficient − 1.11, 95% CI (− 1.96, − 0.27)] of Group 4 was significantly reduced (p = 0.010).

**Table 3:** The association between BMI status and IVF treatment outcomes

	Model 1		Model 2	
	β-coefficient (95% CI)	P-value	β-coefficient (95% CI)	P-value
Available embryo rate (%)				
Group 1	Reference		Reference	
Group 2	0.91 (− 2.35, 0.54)	0.218	− 1.32 (− 2.53, 0.12)	**0.031**
Group 3	− 0.04 (− 1.09, 1.00)	0.933	− 0.14 (− 1.01, 0.72)	0.743
Group 4	− 1.26 (− 2.51, 0.01)	**0.048**	− 1.70 (− 2.75, 0.64)	**0.002**
P_-trend_	− 0.27 (− 0.65, 0.12)	0.172	− 0.35 (− 0.67, 0.03)	**0.031**
High-quality embryo rate (%)				
Group 1	Reference		Reference	
Group 2	− 0.79 (− 2.39, 0.81)	0.332	− 0.96 (− 1.92, 0.01)	0.053
Group 3	0.50 (− 0.66, 1.66)	0.397	− 0.01 (− 0.70, 0.68)	0.985
Group 4	− 0.53 (− 1.91, 0.86)	0.454	− 1.11 (− 1.96, 0.27)	**0.010**
P_-trend_	− 0.01 (− 0.44, 0.42)	0.967	− 0.21 (− 0.47, 0.05)	0.107
Fertilization rate (%)				
Group 1	Reference		Reference	
Group 2	− 2.52 (− 3.62, 1.42)	** < 0.0001**	− 2.46 (− 3.50, 1.42)	** < 0.0001**
Group 3	0.24 (− 0.56, 1.03)	0.559	0.23 (− 0.52, 0.97)	0.550
Group 4	− 1.53 (− 2.48, 0.59)	**0.002**	− 1.48 (− 2.39, 0.57)	**0.001**
P_-trend_	− 0.21 (− 0.51, 0.08)	0.153	− 0.18 (− 0.46, 0.10)	0.208
Normal fertilization rate (%)				
Group 1	Reference		Reference	
Group 2	− 2.75 (− 4.02, 1.49)	** < 0.0001**	− 2.91 (− 4.00, 1.83)	** < 0.0001**
Group 3	0.06 (− 0.86, 0.98)	0.896	0.06 (− 0.72, 0.83)	0.888
Group 4	− 2.09 (− 3.19, 1.00)	** < 0.0001**	− 2.24 (− 3.18, 1.29)	** < 0.0001**
P_-trend_	− 0.37 (− 0.70, 0.03)	**0.034**	− 0.36 (− 0.65, 0.07)	**0.014**
Cleavage rate (%)				
Group 1	Reference		Reference	
Group 2	0.01 (− 0.29, 0.31)	0.966	0.02 (− 0.28, 0.33)	0.883
Group 3	0.21 (− 0.01,0.43)	0.058	0.22 (0.01, 0.44)	0.050
Group 4	− 0.07 (− 0.33, 0.19)	0.599	− 0.04 (− 0.31, 0.22)	0.741
P_-trend_	0.02 (− 0.06, 0.10)	0.672	0.03 (− 0.05, 0.11)	0.514
Blastocyst rate (%)				
Group 1	Reference		Reference	
Group 2	1.63 (− 0.40, 3.66)	0.116	1.27 (− 0.78, 3.32)	0.225
Group 3	1.87 (0.39, 3.35)	**0.013**	1.37 (− 0.10, 2.84)	0.068
Group 4	0.95 (− 0.81, 2.71)	0.289	0.11 (− 1.68, 1.90)	0.903
P_-trend_	0.46 (− 0.08, 1.00)	0.098	0.20 (− 0.35, 0.74)	0.480
	OR (95% CI)	P-value	OR (95% CI)	P-value
Clinical pregnancy				
Group 1	Reference		Reference	
Group 2	1.21 (1.07, 1.38)	**0.003**	0.94 (0.82, 1.07)	0.329
Group 3	1.04 (0.95, 1.14)	0.405	1.00 (0.91, 1.11)	0.926
Group 4	1.18 (1.05, 1.31)	**0.004**	0.91 (0.81, 1.03)	0.131
P_-trend_	1.04 (1.00, 1.07)	**0.037**	0.98 (0.95, 1.02)	0.349
Abortion				
Group 1	Reference		Reference	
Group 2	1.32 (1.00, 1.74)	**0.048**	1.07 (0.80, 1.42)	0.648
Group 3	0.87 (0.70, 1.09)	0.240	0.85 (0.68, 1.07)	0.164
Group 4	1.26 (0.99, 1.61)	0.060	1.02 (0.80, 1.32)	0.858
P_-trend_	1.03 (0.95, 1.11)	0.508	0.98 (0.90, 1.06)	0.593
Live birth				
Group 1	Reference		Reference	
Group 2	1.18 (1.03, 1.34)	**0.016**	0.93 (0.81, 1.07)	0.305
Group 3	1.07 (0.97, 1.18)	0.153	1.04 (0.94, 1.15)	0.437
Group 4	1.13 (1.01, 1.27)	**0.032**	0.91 (0.80, 1.02)	0.105
P_-trend_	1.03 (1.00, 1.07)	0.061	0.99 (0.95, 1.02)	0.484

Model 1 adjusted for female age and male age

Model 2 adjusted for female age, male age, duration of infertility, E2 on HCG day, FSH on HCG day, number of available embryos, number of high-quality embryos, and left testicular volume.* P* < 0.05 was considered statistically significant and was marked in bold

## Discussion

This is the largest study to date evaluating the individual and combined effects of male and female BMI on embryonic development and the CPR, LBR, and abortion rates after IVF. Our research indicated that being overweight/obese reduces the number of available embryos and high-quality embryos among couples undergoing IVF treatment and adversely affects the available embryo rate, high-quality embryo rate, and normal fertilization rate, with sex differences. Therefore, overweight/obesity may affect embryo formation and early development. There is evidence that obesity is associated with an increased risk of infertility and adverse pregnancy outcomes [15, 26, 27]. The meta-analysis report also stated that women with increased BMI have worse IVF outcomes than women with a normal BMI, namely, higher cycle cancellation rates, fewer harvested oocytes, fewer available embryos, and lower LBRs, and the number of abortions among women with a BMI ≥ 30 kg/m2 is also increased (OR 1.52, 95% CI 1.28–1.81, p < 0.001) [14, 28–31]. However, these studies are affected by confounding factors such as the ages of members of the couple, the number of IVF–ET cycles, male BMI, a small sample size, differences in BMI standards by race and the number of harvested oocytes, and these confounding variables do affect the accuracy of evaluations of pregnancy outcomes. Our study fully considered these confounding variables. Previous studies have shown that the number of aspirated oocytes is a predictor for IVF treatment outcomes [32, 33]. The harvesting of too few oocytes affects not only the number of available embryos but also the quality of the oocytes and embryos, which then affects the IVF outcome [34]. Therefore, we excluded patients with fewer than 4 harvested oocytes, and in the general data analysis, we further compared the number of harvested oocytes among the four groups and found no statistically significant difference (P = 0.059).

To reduce the impact of multiple transplant cycles on the results of IVF, we assessed only the first fresh transplant cycle. Even when we restricted the ages of both spouses to between 22 and 36 years, the baseline distribution of the variables, including age, among the four groups was still unbalanced, and we further adjusted for the ages of both spouses. After excluding these confounding factors, our research indicated that overweight/obesity does not affect the CPR, LBR or abortion rate after IVF. This is similar to the results of Petersen et al. [25], who showed that compared with normal weight, neither individual nor combined overweight/obesity significantly reduces the LBR after IVF. In addition, Xie et al. analysed the relationship between prepregnancy BMI and pregnancy and the perinatal period in 398,368 Chinese women and concluded that after adjusting for confounding factors such as age, race, educational level, residential area, occupation, smoking status, passive smoking and alcohol consumption, there was no significant association between female prepregnancy BMI and birth defects and miscarriage [35]. At the same time, we found that when the number of harvested oocytes was not less than 4, there was no significant difference in the number of harvested oocytes between the overweight/obesity group and the normal weight group; however, female overweight/obesity significantly reduced the rates of available embryos (adjusted β-coefficient − 1.32, 95% CI (− 2.53, 0.12), p = 0.031), fertilization (adjusted β-coefficient − 2.46, 95% CI (− 3.50, 1.42), p** < 0.001**), and normal fertilization (adjusted β-coefficient − 2.91, 95% CI (− 4.00, 1.83), p** < 0.001**). Combined male and female overweight/obesity also significantly reduced the rate of high-quality embryos (adjusted β-coefficient − 1.11, 95% CI (− 1.96, 0.27), p = 0.010), and the individual female and combined male and female overweight/obesity groups had much lower numbers of available embryos and high-quality embryos (p < 0.05).

Increasing attention has been given to the impact of male BMI on the outcomes of IVF. Schliep suggested that the BMI of both males and females (rather than one party) jointly affects the pregnancy success rate of IVF treatment [21]. Anifandis suggested that male BMI has a greater impact on embryo quality than female BMI [24]. However, our research indicated that male overweight/obesity had no adverse effects on the numbers of available embryos or high-quality embryos and no significant adverse effects on other IVF outcomes. This may be related to the fact that we controlled for a male age of 36 years, as the increase in male age increases the sperm DNA fragmentation index (DFI) and reduces the normal morphological rate [36, 37]. A number of studies have shown that increased sperm DFI can affect embryonic development and even increase the partner's abortion rate [38, 39]. Regarding the effect of male BMI on the LBR and CPR, our research results are consistent with the conclusions of Le and Schliep [21, 40], namely, that compared with normal male BMI, increased male BMI has no significant effect on the CPR and LBR after IVF (p > 0.05). In contrast, the results of Anfandis’s study [24] suggest that compared with overweight men, regardless of their partner’s BMI, the CPR of normal-weight men is increased; however, the sample size in these studies was small, and important potential confounding factors, such as male and female age, that might affect the conclusion were not considered. Previous studies have shown that age and sex hormone levels affect fertility [41]. A recent meta-analysis showed that an increased male BMI significantly reduced the CPR and LBR after ART (P < 0.05) [23], which might further affect the sperm DFI by affecting changes in sex hormone parameters. In our initial baseline data, there were significant differences in age and sex hormone levels that might further affect sperm DFI by affecting changes in sex hormone parameters. In our initial baseline data, there were significant differences in age and sex hormone levels (FSH days) among the men and women in each group (p < 0.05). To reduce the effects of these factors, we established an adjustment model for age and other influencing factors. After these factors were excluded, our conclusions were more reliable.

Our study has several advantages, including its relatively large sample size, prospective evaluation, consideration of the combined impact of male and female BMI, use of standardized methods to measure BMI, full consideration of confounding factors affecting IVF outcomes and establishment of an adjustment model. Nevertheless, our research still has some limitations. Due to the unavailability of relevant data from other IVF centres in China, the research data are limited to our research centre. It is recommended that future research include more effective and reliable multicentre research data and further explore the influence of overweight and obesity on IVF outcomes. Additionally, the influence of mental health on IVF outcomes has increasingly become a concern. The decision to undergo IVF is a significant financial and emotional investment. The fear of not being able to conceive or of a failed pregnancy can place considerable pressure on couples. However, current research suggests that stress levels and mental health may affect IVF outcomes [42, 43]. Future research should consider the mental health of IVF-ET patients as a confounding factor. In addition, due to energy and time constraints, although we measured male and female BMI in strict accordance with the Chinese BMI standards, we did not distinguish the degree of obesity, nor did we compare the quality of M2 oocytes and blastocysts. Future work is needed to evaluate these confounding factors. Finally, our study indicated that there was no significant difference in smoking status among the four groups, but other lifestyle factors in the entire sample, such as alcohol consumption, could not be considered. However, the impact of alcohol consumption on IVF outcomes is controversial [44, 45]. In our other related studies, we found that the number of people who drank alcohol in the included population was very small. Thus, we do not think that alcohol consumption is the main source of bias in our research. In any case, given that alcohol consumption may adversely affect IVF results [46], future studies should include valid and reliable measurements of alcohol consumption, in addition to other confounding lifestyle factors, when assessing the effect of BMI on IVF outcomes.

## Conclusion

In summary, this study clearly identified the negative effects of overweight/obesity on the fertilization rate and embryo quality after IVF treatment. In addition, there are sex differences. However, a larger, well-designed study is needed to verify the results obtained in this study. It is recommended that future studies obtain more data from other domestic IVF centres and try to explore the mechanism of the influence of BMI on embryo quality and the fertilization rate.

## Data Availability

The datasets used and/or analysed during the current study are available from the corresponding author on reasonable request.
